# Understanding pandemic resilience: a mixed-methods exploration of burdens, resources, and determinants of good or poor well-being among Austrian psychotherapists

**DOI:** 10.3389/fpubh.2023.1216833

**Published:** 2023-08-24

**Authors:** Yvonne Schaffler, Monika Bauer, Barbara Schein, Andrea Jesser, Thomas Probst, Christoph Pieh, Elke Humer

**Affiliations:** ^1^Department for Psychosomatic Medicine and Psychotherapy, University for Continuing Education Krems, Krems, Austria; ^2^Division of Psychotherapy, Department of Psychology, Paris Lodron University Salzburg, Salzburg, Austria; ^3^Faculty of Psychotherapy Science, Sigmund Freud University Vienna, Vienna, Austria

**Keywords:** COVID-19 pandemic, well-being (WHO-5), psychotherapists, resources, qualitative content analysis, coping strategies, physical activity

## Abstract

**Introduction:**

The COVID-19 pandemic has exacerbated the mental health burden on the general population, resulting in increased demands on mental healthcare professionals, including psychotherapists. This cross-sectional study assessed the challenges and resources encountered by 513 psychotherapists based on an online survey conducted between April and May 2022.

**Methods:**

Qualitative methods content analysis of written reports was employed to investigate the emerging challenges and sources of support during the pandemic. A comparative analysis of burdens, resources, sociodemographic factors and daily physical activity was conducted to discern patterns of good and poor well-being.

**Results:**

The predominant burden identified was mental health-related issues, followed by global crises and government-imposed restrictions to mitigate virus transmission. Essential resources encompassed social connections, mindfulness, work satisfaction, and internal processes. Notably, psychotherapists demonstrating good well-being were older, more physically active, had a lower proportion of females, were employed in private practices rather than in institutionalized settings, had more years of professional experience and treated more patients weekly than their counterparts with poor well-being. Furthermore, they exhibited greater optimism, health focus, and satisfaction with their coping methods.

**Discussion:**

These findings can help develop support systems, policies, and educational programs to better support mental health professionals during global crises and offer strategies for individual practitioners to maintain their well-being.

## 1. Introduction

The COVID-19 pandemic has profoundly impacted society, affecting citizens’ physical and mental health due to the disease itself and the implementation of various measures, such as lockdowns and social distancing (1–5). Austria implemented its inaugural lockdown on March 16, 2020, imposing stringent measures that remained in place until May 1, 2020. Subsequently, a second wave of COVID-19 brought about a second lockdown from November 17 to December 6, 2020, followed by a third lockdown from December 26 to January 24, 2021. The emergence of the Alpha and Delta strains of SARS-CoV-2 caused a resurgence in cases in March and April 2021. Another general lockdown was prompted on November 22, 2021. As the government aimed to encourage more people to get vaccinated, not vaccinated people had to abstain from movement and social gatherings already earlier, namely from November 15, 2021. While vaccinated, individuals were released from this lockdown on December 13, 2021; unvaccinated individuals had to wait until January 31, 2022, to be released from the restrictions. On February 5, 2022, a mandate requiring vaccination was implemented, but it was rescinded on July 29, 2022 (6).

Already the initial lockdown in Austria resulted in a surge of mental health concerns, including depression (20%), anxiety disorders (19%), and insomnia (16%) among the general population (2) Subsequent studies revealed that these effects persisted beyond the lockdowns (3, 7). The increasing prevalence of mental illnesses in the general population and the growing need for professional mental health services (8, 9) underscores psychotherapists’ importance in providing optimal care to individuals struggling with mental health issues. The role of psychotherapists in addressing mental health concerns cannot be overstated (10). Nonetheless, the success of psychotherapy is inextricably tied to the mental state of psychotherapists. Exhausted psychotherapists may resort to distancing themselves or becoming emotionally disconnected from their clients to conserve their limited energy, leading to a decline in favorable client outcomes and a diminution in the therapist’s sense of gratification derived from therapeutic work (11). Thus, the question arises about the burden of psychotherapists and how they deal with situations of prolonged strain, given that they are also susceptible to the pandemic and its ramifications on mental well-being (12).

Research has indicated that the COVID-19 pandemic has substantially impacted the well-being of frontline healthcare professionals across the world. This is exemplified by the outcomes of various meta-analyses, highlighting increased anxiety, depression, sleep disorders, and burnout among physicians and nurses (13–15). However, there is a dearth of empirical evidence regarding the effects of the pandemic on other healthcare professions, such as psychotherapists (16). A study of 1,500 psychotherapists in Austria at the beginning of the pandemic found that their stress levels were higher than those in the general population prior to the pandemic (17). Factors contributing to heightened stress levels among psychotherapists included concerns regarding infection during direct patient contact, adjustments in day-to-day practices such as transitioning to remote therapy and working while wearing masks, managing increased demand for treatment and waitlists, and modifications in patient symptoms (18). More recent research in Austria showed that Austrian psychotherapists manifested better psychological health than the general population during the COVID-19 outbreak (16). Notwithstanding the comparatively favorable psychological well-being of psychotherapists as compared to the general population, a substantial proportion of them surpassed the threshold for clinically significant insomnia (5%), depression (11%), anxiety (11%), and stress (37%) (16). The escalating need for therapeutic aid and the heightened psychological strain experienced by patients because of COVID-19-related restrictions, illness anxiety, unemployment, economic downturns, and societal fluctuations pose a severe challenge (19, 20), which has led to a surge in burnout among mental health professionals (21, 22). However, the specific burdens reported by psychotherapists and their relative magnitude have yet to be determined.

Moreover, further research is required to decipher why psychotherapists, despite this confluence of strains, still show lower odds of exceeding cut-offs for clinically relevant depression (aOR 0.41; 95% CI: 0.29, 0.57), anxiety (aOR 0.58; 95% CI: 0.40, 0.83), insomnia (aOR 0.51; 95% CI: 0.31, 0.83) and moderate to high stress levels (aOR 0.34; 95% CI: 0.26, 0.44) compared to the general population. Previously assumed factors contributing to this relative resilience include high professional motivation, a secure social background and the possibility of independent time management since most are self-employed (16).

It has been found that both psychotherapists and clinical psychologists in Austria experienced better mental health than the general population during the COVID-19 pandemic (23). Through a qualitative exploration of their self-reported strains and resources, it was discovered that these professionals possessed a heightened awareness of pandemic-related mental health issues and adeptly employed adaptive coping strategies to address them (24). Also, in a Brazilian study, the resilience of psychologists during the pandemic has been attributed to their training and mastery of adaptive measures (25). Consequently, it is crucial to investigate the specific coping methods employed by psychotherapists in the face of enduring crises, given their unique expertise in navigating such challenges. This article defines coping as the active engagement with one’s internal and external resources to alleviate distress.

The stress level experienced by mental health professionals can significantly impact their approach to coping with challenging situations and vice versa. Research has demonstrated that avoidant coping strategies (such as denial, distraction, and substance use) are linked to heightened stress levels, leading to decreased overall well-being. Conversely, active coping strategies, such as maintaining a positive attitude, problem-solving, and seeking social support, positively impact well-being and are inversely related to psychological distress (26–29). In this regard, various resources have been suggested, including engaging in physical activity, incorporating relaxation techniques into the work routine, participating in mindfulness-based resilience training programs, and practicing autogenic training (30). Regular physical activity has been emphasized for its efficacy in preventing and ameliorating specific psychological disorders, such as depression (31–33). Evidence backs the positive impact of self-care routines, including awareness, balance, physical health and social support in decreasing negative outcomes such as burnout or professional impairment among mental health professionals (34).

Our study, conducted in April and May 2022, seeks to augment the current body of research on the mental well-being of psychotherapists throughout the pandemic. Our principal objective is to examine self-reported burdens and the resources psychotherapists rely on to manage distress. Additionally, we aim to shed light on group variances in self-reported burdens and resources anchored on individuals’ well-being status 2 years into the pandemic. To comprehensively understand each group’s attributes, we also conduct a comparative study of sociodemographic factors, including the participants’ work setting and physical activity levels. We consider psychotherapists’ work setting because previous studies indicated that self-employed individuals generally experience higher job satisfaction than their salaried counterparts, largely attributable to increased autonomy, flexibility, and effective skills utilization (35, 36). On the contrary, employed health personnel often face high-stress levels related to rigid, changing protocols, a heavy workload, and a sense of not being valued (35, 36). The advantages of self-employment have been posited as one factor to explain the better mental health of psychotherapists compared to the broader Austrian population, as most psychotherapists in Austria operate self-employed in private practice (16). Hence, working in private practice might correlate with enhanced well-being among psychotherapists. Furthermore, given previous studies associating increased physical activity with better mental health during the pandemic (2, 37), we also assess our participants’ physical activity levels to test the assumption that high physical activity correlates with good well-being in this group.

## 2. Methods

### 2.1. Design

From April 11 to May 31, 2022, we conducted a cross-sectional internet-based survey using Research Electronic Data Capture (REDCap; Vanderbilt University, Nashville, TN, USA) (38). The presented study is a sub-study of a more extensive survey that included 49 items. It was distributed via email to psychotherapists registered with the Austrian Federal Ministry of Social Affairs, Health, Care and Consumer Protection (>11,000 psychotherapists registered in April 2022), providing a valid email address (≈7,000 psychotherapists) (16), and to clinical psychologists registered in the list of the Austrian Federal Ministry of Social Affairs, Health, Care and Consumer Protection (>11,000 clinical psychologists registered in April 2022) (23, 24). Before conducting the study, it was approved by the data protection officer and Ethics Committee of the University for Continuing Education Krems, Austria (Ethical number: E.K. G.Z. 11/2021–2024). All participating psychotherapists provided electronic informed consent. Participation was entirely voluntary and uncompensated.

### 2.2. Measures

#### 2.2.1. Sociodemographic variables

Participants were inquired about their gender, age, years of professional experience, form of employment (private practice, institution), and their level of physical activity. Per previous studies (39, 40), physical activity was assessed by asking how many days per week participants engaged in physical activity of at least 60 min in numerical response.

#### 2.2.2. Open-ended questions on perceived burdens and resources

To assess the challenges and resources experienced by psychotherapists during the ongoing crises, the survey included five open-ended questions (1–5) and one structured question (6):

1. What are your primary current sources of burden?
2. How are these burdens manifesting themselves at present?
3. Looking back on the past 2 years, what impacts have you observed of the pandemic on your mental health and well-being?
4. What strategies have you employed to manage the adverse impacts of the pandemic?
5. Have there been any positive impacts resulting from the pandemic as well?

For questions 1–5, respondents were provided with an open-ended response format. They were allowed to describe their personal experiences in their own words, ranging from single-word answers to lengthy paragraphs. Respondents also had the option to leave the answer field blank and skip any of the free-text questions.

#### 2.2.3. Structured question on resources

1. On a scale of 1 to 5, how satisfied are you with your implemented coping strategies? Please rate your level of satisfaction as follows: 1 - very satisfied, 2 - satisfied, 3 - neutral, 4 - dissatisfied, 5 - very dissatisfied.

For question 6, respondents were given a numerical response format, encouraging them to express their answers using a designated value. The participants had to understand that question 6 was related to question 4.

All questions and free-text answers were initially formulated in German.

#### 2.2.4. Well-being (WHO-5)

We employed the 5-item World Health Organization Well-being Index (WHO-5) (39) to gauge the well-being of participants. This index comprises five questions that capture positive aspects of well-being over the past 2 weeks, focusing on positive mood (such as feeling relaxed or in good spirits), vitality (such as waking up feeling refreshed and being physically active), and general interest (such as feeling interested in things). Participants rated their responses on a six-point Likert scale ranging from 0 (never) to 5 (all the time). The raw score ranges from 0 to 25, with higher scores indicating greater well-being. To transform the score into a percentage scale ranging from 0 (indicating an absence of well-being) to 100 (indicating optimal well-being), we multiplied the scores by 4, as recommended by previous studies (40). Cronbach’s alpha was α = 0.85 in the present sample.

### 2.3. Analyses

#### 2.3.1. Content analysis

The qualitative data obtained from the open-ended questions were analyzed using conventional qualitative content analysis (41) by two coders (MB and BS), followed by quantifying the qualitative categories. Specifically, our approach aligns with Udo Kuckartz’s approach to content analysis, which includes initial data assessment, thematic categorization, multiple coder analysis, iterative category refinement, use of software for coding, detailed codebook creation, and final identification of distinct themes and subthemes (42).

Following an initial data assessment, the research team determined that collectively analyzing the responses to questions 1–3 and 4–6 would be the most effective approach. The former questions could be thematically grouped as psychotherapists’ burdens, while the latter pertained to their resources. For questions 1–3 (burdens), 167 questions remained unanswered. Thirty nine participants did not answer all three questions, 12 did not answer two questions, and 26 did not answer one. For questions 3–6 (resources), 125 questions were ignored. One participant did not answer all three questions, 39 did not answer two and 44 missed only one. Respondents could mention various aspects in their responses so that multiple categories could be assigned to each answer. The number of codings per case (*N* = 513) for questions 1–3 on burdens varied between 1 to 12, while for questions 3–6 regarding resources, it ranged from 1 to 16. The written reports ranged from being multiple lines in length with nuanced expressions to being presented as brief bullet points.

The first coder (MB) analyzed the responses to questions 1–3 regarding the burdens, while the second (BS) coder focused on questions 4–6 regarding resources. Both coders familiarized themselves with the data by reading it thoroughly before categorizing it. The first author (YS) supervised the coding process, providing the coders with a deductive code list from a parallel similarly designed study on burdens and resources of Austrian clinical psychologists (24). The assumption was that both groups experienced comparable burdens. The parallel research relied on the same open-ended questions posed to the participants, except that it did not include question 6 (aiming at a 1–5 rating of satisfaction with one’s coping strategies). We used this code list as a starting point, checked whether the same categories were present in our data, and made modifications where necessary. Eventually, we inductively found five additional subcategories for questions 1–5 and five numerical categories for the additional question six. Question six was analyzed with the same software for qualitative data. Category definitions, coding rules, and examples were documented in a codebook.

**Figure 1:**
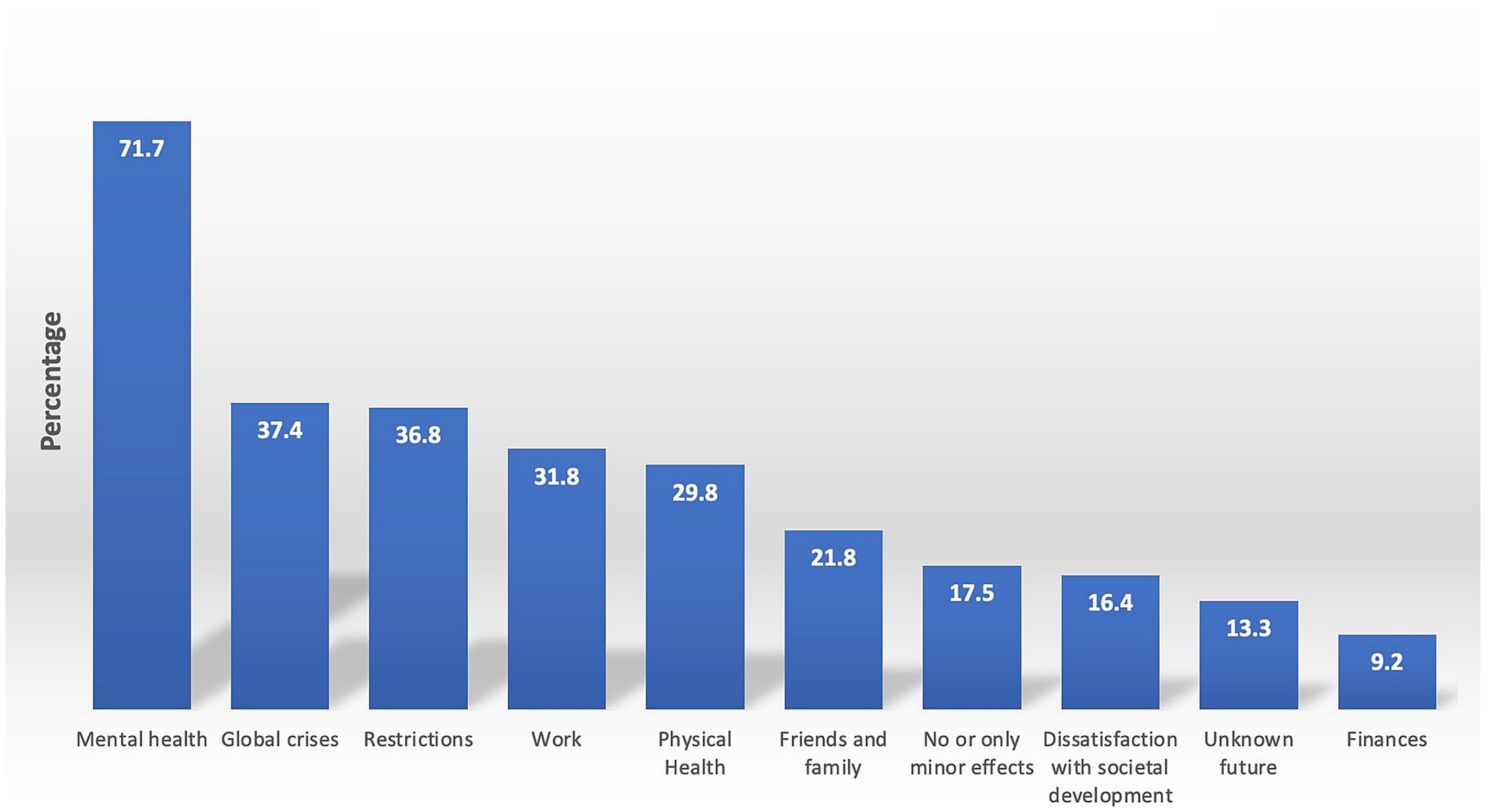
Burdens among psychotherapists. Presented are the results of a qualitative content analysis of answers to questions 1–3 in per cent, inquiring about the current burdens experienced by psychotherapists (question 1), how these burdens currently manifest (question 2), and what impacts of the pandemic on mental health and well-being have been observed when looking back at the last 2 years (question 3). The percentages of participants reporting one or more burdens in each of the main categories are displayed, with the understanding that the percentages of the main categories may differ from the sum of the percentages in the individual subcategories due to the possibility of a respondent reporting experiences in multiple subcategories within a single main category. For instance, a respondent may have reported being burdened by the Russian-Ukraine war and climate crisis, resulting in their appearance in each subcategory but only being counted once per main category.

**Figure 2:**
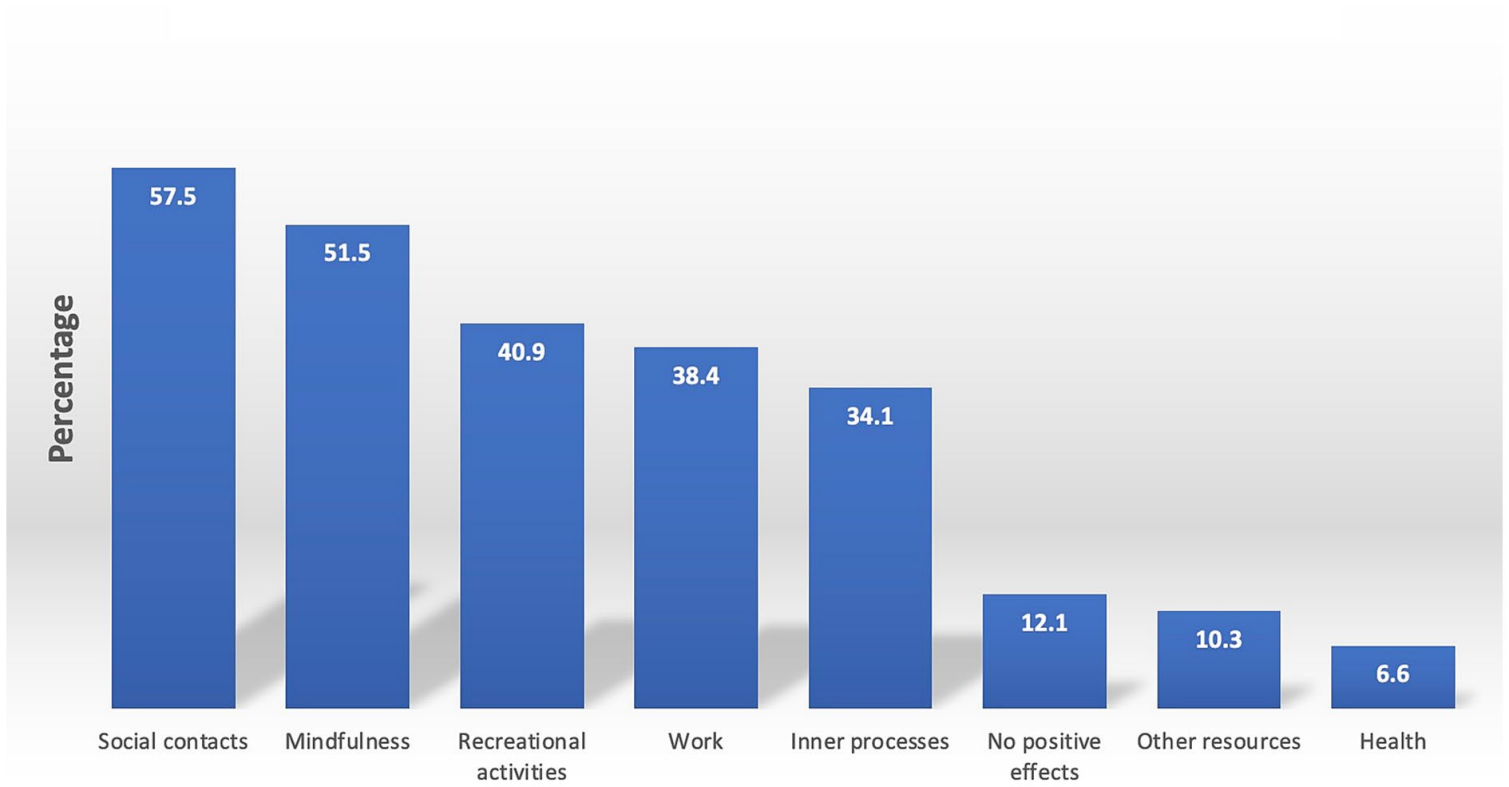
Resources psychotherapists have accessed. Presented are the results of a qualitative content analysis of answers to questions 4–5 in per cent, inquiring about what helped psychotherapists cope with the pandemic’s adverse impacts (question 4) and whether they observed any positive impacts of the pandemic when looking back at the last 2 years (question 5). The percentages of the main categories may not add to the sum of the percentages in the individual subcategories described in the following sections, as some respondents may have reported multiple subcategories within a single main category. For instance, a respondent may have reported engaging in hobbies and physical activity as recreational activities, resulting in their appearance in each subcategory but only being counted once per main category.

**Figure 3:**
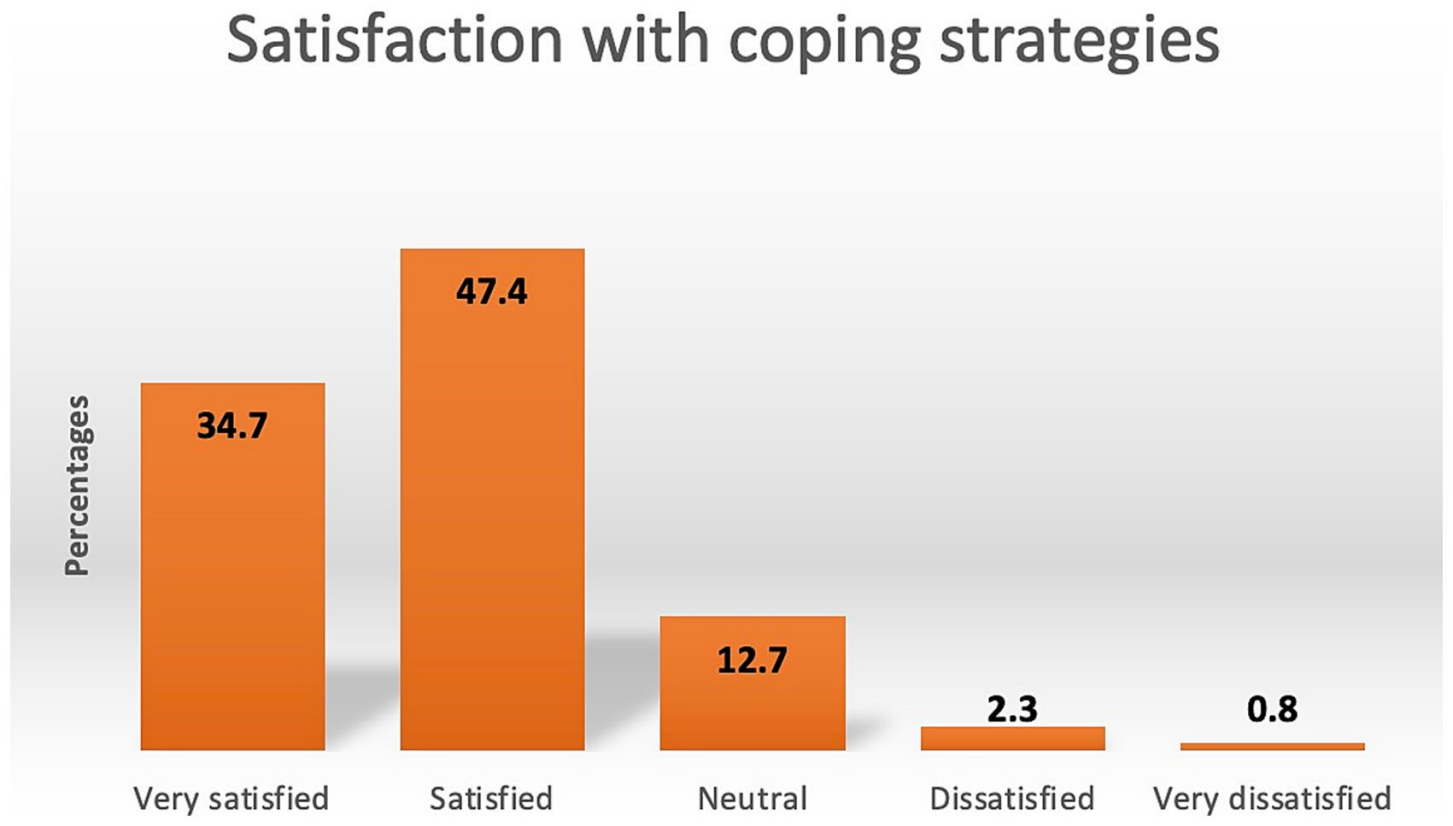
Satisfaction with coping strategies psychotherapists accessed to deal with burdens. The percentages of respondents reporting one designated value (1 – very satisfied, 2 – satisfied, 3 – neutral, 4 – dissatisfied, 5 – very dissatisfied) resulted from the statistical analysis of question 6: Question 6 asked respondents. “How satisfied are you with the coping strategies you have implemented?”.

The coders used the ATLAS.ti vers. 23.1.0 (43) software to code their respective datasets according to their list of categories. After coding 10% of cases by the coders and the supervisor separately, mismatching codings per case were discussed within the team of coders, and slight adaptations were made to the category systems. After coding the entire dataset, the coders and the supervisor read all quotations assigned to each category to correct coding errors. The data analysis resulted in 10 categories (Figure 1) with 22 subcategories for the burdens of psychotherapists (Questions 1–3) and eight categories (Figure 2) with 30 subcategories for their resources (Questions 4–5). Question six yielded five categories (Figure 3).

#### 2.3.2. Across-group comparison

To conduct a comparative analysis of sociodemographic factors, physical activity, burdens and accessed resources according to groups, we partitioned our sample based on their levels of psychological well-being, following de Girolamo et al. (44). They used the WHO 5-item well-being scale (WHO-5) to identify profiles of individuals with varying well-being levels. Specifically, individuals with a WHO-5 score of 51–100 were assigned to the “Good WB” group, denoting good well-being (n = 342). In contrast, those with a score of ≤50 were placed in the “Poor WB” group, suggesting a need for further investigation of potential depression symptoms (*n* = 171) (40). The groups were defined as document groups within the Atlas.ti software. To compare the coding scores of each group, we utilized the Atlas.ti cross-tabulation function (Code-Document Analysis), which enabled us to compare the distribution of codings for each category per group. Chi-square tests were applied using SPSS version 26 (IBM Corp., Armonk, NY, USA) to analyze differences in the sample attributes and the number of codings per group. Potential differences in answers on how many days each group engaged in physical activity were assessed with *t*-tests for independent samples. *p*-values of less than 0.05 indicate statistically significant differences (two-tailed tests).

## 3. Results

### 3.1. Sample characteristics

530 psychotherapists participated in the survey, yielding a response rate of approximately 7.6%. Of these, 513 completed all outcome variables, resulting in a completion rate of 96.8%. The analysis considered only psychotherapists with complete data. Detailed characterization of the sample compared to the total sample of all licensed Austrian psychotherapists is provided in our companion paper (16). In brief, female psychotherapists, those with a humanistic orientation and fewer years in the profession, were overrepresented.

Table 1 provides a detailed account of the sociodemographic and professional characteristics of WB-group differences. The average age of the cohort was 53.06 ± 9.94 years, with a significant majority (80.5%) identifying as female. Within the “Poor WB” group, a considerably higher proportion of participants were female (86.0%) compared to the “Good WB” group (77.8%; *p* = 0.027). The “Good WB” group, on the other hand, displayed a higher percentage of older individuals (60–69 and 70+ years), whereas the “Poor WB” group comprised more participants aged 41–49 and 50–59. Both groups exhibited a similar percentage of individuals under 40 (*p* = 0.038). Additionally, the “Good WB” group encountered more weekly patients (19.13, SD = 9.27) than the “Poor WB” group (16.98, SD = 8.88; *p* = 0.013). A more significant percentage of the “Good WB” group operated exclusively in private practice (80.4%) compared to the “Poor WB” group (71.9%). Conversely, significantly more psychotherapists in the “Poor WB” group were employed by institutions (28.1%) as opposed to the “Good WB” group (19.6%; *p* = 0.030). Physical activity patterns differed between groups, with the “Good WB” group participating more frequently (4+ days/week) and the “Poor WB” group less regularly (0–2 days/week; *p* = 0.001).

**Table 1:** Study sample characteristics (*N* = 513).

	Group		
Variable	Good WB (*n* = 342)	Poor WB (*n* = 171)	Statistics
Gender			
Female, % (*N*)	77.8 (266)	86.0 (147)	χ^2^ (1) = 4.869;
Male, % (*N*)	22.2 (76)	14.0 (24)	*p* = 0.027
Age in years, M (SD)	53.84 (10.24)	51.51 (9.13)	t (511) = −2.521; *p* = 0.012
Age			
<40, % (*N*)	8.5 (29)	9.4 (16)	χ^2^ (4) = 10.156; *p* = 0.038
41–49, % (*N*)	25.4 (87)	31.0 (53)	
50–59, % (*N*)	37.1 (127)	43.3 (74)	
60–69, % (*N*)	21.9 (75)	13.5 (23)	
≥70, % (*N*)	7.0 (24)	2.9 (5)	
Professional experience in years, M (SD)	12.99 (10.09)	11.22 (9.42)	t (500) = −1.882; *p* = 0.060
Number of patients per week, M (SD)	19.13 (9.27)	16.98 (8.88)	t (508) = −2.506; *p* = 0.013
Form of employment as psychotherapist			
Solely private practice, % (*N*)	80.4 (275)	71.9 (123)	χ^2^ (1) = 4.713; *p* = 0.030
Institution, % (*N*)	19.6 (67)	28.1 (48)	
Physical activity for at least 60 min/per day			
0 d/wk., % (*N*)	5.6 (19)	14.6 (25)	χ^2^ (7) = 24.477; *p* = 0.001
1 d/wk., % (*N*)	12.9 (44)	11.7 (20)	
2 d/wk., % (*N*)	14.0 (48)	22.2 (38)	
3 d/wk., % (*N*)	19.9 (68)	20.5 (35)	
4 d/wk., % (*N*)	14.0 (48)	6.4 (11)	
5 d/wk., % (*N*)	11.7 (40)	8.8 (15)	
6 d/wk., % (*N*)	7.9 (27)	5.3 (9)	
7 d/wk., % (*N*)	14.0 (48)	10.5 (18)	

### 3.2. Comprehensive group burdens

Inquiring about their burdens, psychotherapists were presented with three open-ended questions. Qualitative content analysis showed that psychotherapists in Austria face numerous challenges, including mental health, global crises, COVID-19 restrictions, work, physical health, dissatisfaction with societal development, finances, and uncertainty about the future. Only a minority of participants (17.5% or *N* = 90) reported experiencing no or only minor adverse impacts. The results are described in more detail, starting with the largest category.

#### 3.2.1. Mental health

71.7% of respondents (*N* = 368) reported burdens related to mental health, which can be further categorized into seven subcategories. Among psychotherapists, negative feelings such as irritability, impatience, disappointment, or despair were identified as the most prevalent burden, accounting for 34.9% (*n* = 179). For example, one respondent stated having “*felt high levels of inner aggression due to the sometimes pointless seeming measures*.” 18.9% (*n* = 97) of psychotherapists reported symptoms of exhaustion, including reduced resilience, fatigue, or less energy. Excessive demand was also a common issue, with 18.5% (*n* = 95) of respondents describing not having enough time, having difficulty managing their resources, or worrying about being unable to meet the required workload. One participant said, “*I often felt confined to work alone and isolated from the other facets of life*.” 16.4% (*n* = 84) of respondents identified rumination as a burden and described constant worrying and repetitive thoughts. 12.7% (*n* = 65) mentioned problems with sleep, such as difficulty falling asleep or staying asleep, shortened sleep duration, or poor sleep quality. Depressive mood was mentioned by 4% (*n* = 21), and only 2% (*n* = 10) expressed concern about the mental health of a friend or family member.

#### 3.2.2. Global crises

A significant proportion of the respondents, precisely 37.4% (*N* = 192), expressed concerns about the repercussions of four major global crises, which they perceived as burdensome. 33.1% (*n* = 170) cited the Russian aggression against Ukraine as a source of worry, using statements like this: “*The war in Europe - the suffering, destruction, and fear of it spreading to other countries (Europe, World War, nuclear weapons)*.” Furthermore, 10.9% (*n* = 56) raised issues regarding the pandemic. 8% (*n* = 41) expressed their apprehension about the climate crisis, and 1% (*n* = 5) expressed worries about the well-being of refugees.

#### 3.2.3. COVID-19 restrictions

An additional concern raised by 36.8% (*N* = 189) pertained to the COVID-19 restrictions. These individuals commented on the various restrictions, including lockdowns, mandatory vaccinations or masks, and the resulting consequences. Limited opportunities for recreational activities and the lack of social interaction were also frequently mentioned. For instance, one respondent said: “*Hugging people behind protective screens or being asked by a policewoman to stand up from a public bench because it is ‘not allowed’ to sit during the pandemic - all of these experiences are disturbing for the psyche*.” On the other hand, the relaxation or absence of restrictions was also viewed negatively, as some respondents felt inadequately protected when meeting people with a critical stance towards vaccinations and protective measures.

#### 3.2.4. Work

31.8% (*N* = 163) of the respondents experienced work-related strain. Of those, 24% (*n* = 123) felt burdened by a high workload, including long working hours and many patient requests. Respondents also expressed concerns about the increased prevalence and complexity of mental disorders among their patients and how they were affected by similar issues. One respondent stated, “*The increasingly challenging conditions of my patients are on my mind more often than usual; I find myself reflecting more on my psychotherapeutic interventions and setting. The themes of my patients overlap more frequently with issues that also affect me personally, resulting in a stronger emotional involvement in my work as a psychotherapist*.”

In addition, 9.8% (*N* = 50) experienced burdens related to their working conditions, such as an unstable working situation, postponed appointments, and too few or too many health insurance positions. Only 1.6% (*n* = 8) experienced burdens related to the workplace atmosphere, such as interpersonal and workplace conflicts within their team. Finally, 1.2% (*n* = 6) felt burdened by the lack of patients.

#### 3.2.5. Physical health

29.8% (*N* = 153) of the participants expressed concerns about their “physical health.” Among them, 23.8% (*n* = 122) reported experiencing “somatic complaints” such as muscle tension, back pain, tinnitus and headaches. Additionally, general concerns, e.g., in the context of ageing, smoking, weight gain or chronic disease, were also expressed. Furthermore, 8.8% (*n* = 45) of the participants reported anxiety about the physical health and mortality of loved ones, including pets.

#### 3.2.6. Other burdens

21.8% (*n* = 112) conveyed concerns related to their social network. 18.1% (*n* = 93) reported interpersonal conflicts, often partnership problems or conflicts related to divergent attitudes toward the COVID-19 containment or vaccination measures. 3.9% (*n* = 20) were troubled by issues concerning their children, such as their progress in school or childcare. Additionally, 16.4% (*n* = 84) of the respondents expressed dissatisfaction with societal development, such as the division of society, poverty or the world situation. Out of these, 9.2% (*n* = 47) were particularly unhappy with the way politics and media reacted to the pandemic: “*I am deeply concerned about the current state of society, which appears to be increasingly radicalized and characterized by inadequate crisis reporting and insufficient attention to the urgent issue of climate change. Also, the potential weakening of democratic values is a troubling development*.” Furthermore, 9.2% (*n* = 47) of the respondents reported financial concerns. Among them, 4.5% (*n* = 23) expressed general worries about their personal financial situation, 3.9% (*n* = 20) were concerned about inflation, and 1.6% (*N* = 8) felt that they were being underpaid. Finally, 13.3% (*n* = 68) indicated worries about the distant future, such as the world’s future, society’s future, or the future of their children or grandchildren.

### 3.3. Comprehensive group resources

After answering inquiries 1–3 about the issue of burdens, psychotherapists were posed with two open-ended questions (4, 5) that focused on exploring the resources and positive outcomes that may have assisted respondents in effectively coping with the burdens above amid the pandemic. Question six aimed at rating satisfaction with one’s resources. The corresponding percentages of the main resource categories are illustrated in Figure 2. Most psychotherapists identified aspects related to social contacts, mindfulness, recreational activities, work, inner processes, other resources and health, and 12.1% (*n* = 62) reported that they had not experienced any positive impacts. Our subsequent description shall explicate the reactions to questions 4–5 in greater detail, commencing with the most salient category, followed by a descriptive statistic of the ratings (question 6).

#### 3.3.1. Social contacts

The category of ‘social contacts,’ which proved to be an essential resource for 57.5% of respondents (*N* = 295), was further divided into five subcategories. Among these, 41.7% (*n* = 214) cited “partners, family, and friends” as a source of support, highlighting the increased opportunity to spend quality time with loved ones and strengthen existing relationships during the pandemic. Additionally, 15.4% (*n* = 79) of respondents mentioned the value of social interactions and conversations in general, while 4.9% (*n* = 25) referred to their colleagues as a source of social support. Interestingly, while social contacts were generally viewed as a positive resource, a small proportion of respondents (7.4%; *n* = 38) expressed relief at having fewer social obligations and options for social withdrawal during the pandemic, such as avoiding gatherings. Finally, 4.3% (*n* = 22) of the respondents identified their pets as an essential source of support.

#### 3.3.2. Mindfulness

Mindfulness practice proved to be a valuable resource for 51.5% (*n* = 264) of respondents. Eight subcategories emerged from their responses. The first, “slowing down,” was mentioned by 22.6% (*n* = 116) of respondents. This category was characterized by a sense of calmness and a reduction in the pace of everyday life. Respondents reported feeling less pressure to be productive during leisure time and, particularly during curfews, retreating from public life and focusing on their private lives.

The second subcategory, “focusing,” was identified by 17.9% (*n* = 92) of respondents. They reported positive impacts such as concentrating on essential matters and “*what you can do and not on what is out of your control*.” While this could be considered a form of mental mindfulness, the respondents did not refer to specific techniques or practices but rather to a sense of serenity resulting from a more conscious way of navigating life.

Another subcategory identified by 13.6% (*n* = 70) of respondents referred to “self-care,” including answers such as “*I cultivate a loving approach towards myself*” or “*I watch out for what nourishes me*.” Mental techniques and exercises such as yoga, breathwork or meditation were embraced by 11.9% (n = 61). 3.7% (*n* = 19) reduced media consumption, 2.7% (*n* = 14) practiced gratitude, 2.3% (n = 12) drew on religion or spirituality, and 1.8% (*n* = 9) practiced acceptance.

#### 3.3.3. Recreational activities

40.9% (*n* = 210) of the sample identified “recreational activities” as a resource. This category consisted of three distinct subcategories: “physical activity,” “being outside,” and “hobbies.” Across all responses, individuals mentioned the impact of the pandemic on their ability to find time for personal pursuits. 23.2% (*n* = 119) of respondents reported engaging in various physical activities such as running, gymnastics, mountain climbing, and other forms of physical activity. The subcategory “being outside” was embraced by 21.8% (*n* = 112). It included activities such as walking, socializing, simply appreciating nature or even “*freeing one’s mind in nature*.” One respondent declared that “*I ‘have to’ go for a walk in the forest daily*.” In addition, 13.5% (*n* = 69) found engaging in a hobby helpful. They referred to reading, gardening, dancing, painting and listening to music. One participant put it like this: “*I engaged with art and culture in the form of books or documentaries, light-hearted films, and music*.”

#### 3.3.4. Work

38.4% (*N* = 197) of respondents reported work-related changes due to the pandemic. This category comprises five subcategories: “flexible working conditions,” “work in itself,” “supervision/intervision,” “less work,” and “recognition of psychosocial services.”

Notably, 19.1% (*n* = 98) of respondents mentioned the “flexible working conditions” subcategory positively, with some reporting the ability to work from home or digitally, saving them time and mental energy. Some respondents recognized benefits for their patients, citing the positive impacts of digital psychotherapy on severely ill patients, including the ability to establish an intensive and positive therapeutic relationship and even provide opportunities for creative approaches. Furthermore, 12.7% (*n* = 65) of respondents regarded their work in general as a resource during the pandemic. Another noteworthy subcategory pertained to attending professional support formats, such as supervision or intervision, which was mentioned by 5.7% (*n* = 29) of respondents. Additionally, 4.5% (*n* = 23) reported decreased clients, appointments, and work commitments, particularly during the curfews implemented during the pandemic’s first year. Finally, it is vital to highlight the increased recognition of psychosocial services, which was positively noted by 2.1% (*n* = 11) of respondents. These individuals observed that the pandemic had brought mental health and psychological disorders to the forefront of professional attention, thereby contributing to the destigmatisation and normalization of seeking psychotherapeutic support.

#### 3.3.5. Inner processes

Another major category, identified by 34.1% (*N* = 175) of respondents, related to inner processes as a resource during the pandemic, such as “a positive attitude,” “resilience,” “self-reflection,” and “resistance.” 15.6% (*n* = 80) of respondents cited their “positive attitude” as a key resource throughout the pandemic. They described focusing on the positive aspects of their situation and looking confidently towards the future. One respondent, for example, reported: “*What helps me above all is my incorrigible optimism and my attitude towards life, my faith and trust in people who live in a solution-oriented way*.” 12.3% (*n* = 63) of respondents referred to their flexibility and adaptability. They identified their courage, emotional stamina, confidence, and competence in handling the pandemic situation or confronting their fears. One respondent said: “*My trust and inner stability, which I have gained through my long-term path of personal development, has helped me greatly*.” Such statements, focusing on personal development as a source for dealing with prolonged crises, were categorized under the subcategory of “resilience.” 8.6% (*n* = 44) of respondents cited self-reflection as a resource, reporting that they confronted their feelings and used the pandemic as an opportunity to develop self-awareness. For example, one respondent described the pandemic as “*training in independent and courageous thinking and decision-making*” Actively reflecting on the pandemic situation also enabled the respondents to develop new perspectives for coping with COVID-19 measures. A mere 2.1% (*n* = 11) attested to adopting a stance of “resistance” against the COVID-19 protective measures that they perceived as capricious and pointless. As one respondent succinctly put it, “*What helped me was the pursuit of information from alternative sources, the conversion of fear into indignation, and the subsequent surge of bravery that spurred me towards action - be it through participation in demonstrations, vocalizing my stance in parliament, taking up political activism and so on*.”

#### 3.3.6. Other resources

10.5% (*N* = 53) said to have relied on “other resources.” 4.7% (*n* = 24) of respondents recognized the benefits of structure, routines, and self-discipline in their personal and professional lives as valuable resources throughout the pandemic. 4.5% (*n* = 23) participants also cited having drawn support from “vacations,” whereas an augmentation in their “financial resources” due to reduced expenses or increased income was reported by 1.4% (*n* = 7).

#### 3.3.7. Health

A favorable consequence of the pandemic, noted by 6.6% (*N* = 34) participants, was an increased focus on “health.” 3.9% (*n* = 20) of respondents reported seeking “professional support for their health,” such as psychotherapy or medical care. 2.7% (*n* = 14) of the respondents expressed an “increased importance of health,” endorsing the implementation of protective measures against illnesses, as one participant said: “*Due to wearing masks, I have had significantly fewer colds and flu-like infections*.”

#### 3.3.8. Satisfaction with coping strategies

After answering the two open-ended questions 4–5 about their resources, psychotherapists were asked to evaluate their satisfaction with the coping strategies they have employed and to express their level of contentment using a five-point scale (question 6). The corresponding percentages of their evaluations are illustrated in Figure 3.

### 3.4. Group characteristics

Supplementary material offers an in-depth overview of the group attributes related to the reported burdens (Supplementary Table 1) and resources (Supplementary Table 2).

#### 3.4.1. Burdens (questions 1–3)

Across all burden-related categories and subcategories, the following exhibited significant differences with more codings in either the Good Well-Being (“Good WB”) or Poor Well-Being (“Poor WB”) group:

In the category of “family and friends,” the subcategory of “interpersonal problems” was significantly more frequently reported by the “Poor WB” group compared to the “Good WB” group (24.6 vs. 15.2%, *p* = 0.010).

In the “mental health” category, the “Poor WB” group reported significantly more “excessive demand” (24.6 vs. 15.5%, *p* = 0.013), “negative feelings” (50.9 vs. 28.7%, *p* = 0.001), problems with “sleep” (18.7 vs. 9.6%, *p* = 0.004), and symptoms of “exhaustion” (33.3 vs. 11.7%, *p* = 0.001) than the “Good WB” group.

In the “physical health” category, the “Poor WB” group reported significantly more physical health burden (46.8 vs. 28.7%, *p* < 0.001) and somatic complaints (40.4 vs. 19.0%, *p* = 0.001) than the “Good WB” group.

No or little adverse impacts were reported by a substantial 26.6% in the “Good WB” group and only 2.9% in the “Poor WB” group (*p* = 0.001).

#### 3.4.2. Resources and satisfaction with coping (questions 4–6)

Across all resource-related categories and subcategories, the following exhibited significant differences with more codings in either the Good Well-Being (“Good WB”) or Poor Well-Being (“Poor WB”) group:

The “mindfulness” category but not any specific subcategories displayed an overall difference, with the “Good WB” group reporting lower levels of mindfulness (78.7 vs. 87.1%, *p* = 0.020).

In the “recreational activities” category, a substantial overall group difference was observed (53.8 vs. 73.1%, *p* < 0.001), with the “Good WB” group likewise reporting fewer recreational activities. Among the subcategories, only the “physical activity” subcategory revealed a significant difference, with the “Good WB” group reporting less frequently that physical activity was a significant resource (20.5 vs. 31.0%, *p* = 0.008).

Conversely, a significant overall difference between the groups was observed in the “inner processes category,” with the “Good WB” group demonstrating more inner processes (43.6 vs. 34.5%, *p* = 0.049). Among the subcategories, solely the subcategory “positive attitude/optimism” revealed a significant difference, as the “Good WB” group reported more frequently sporting a “positive attitude/optimism” (19.6 vs. 10.5%, *p* = 0.009).

No significant difference between the groups emerged within the main category of “health.” However, only members of the “Good WB” group registered in the subcategory “increased importance of health” (4.1 vs. 0%, *p* = 0.007).

Regarding “satisfaction with coping,” the “Good WB” group reported significantly higher levels of satisfaction (*p* < 0.001). In more detail, 43.4% stated to be “very satisfied” with their coping strategies (vs. 19.6% in “Poor WB”), whereas they were less often “neutral” (6.6 vs. 25.6%), dissatisfied (1.2 vs. 4.8%), or very dissatisfied (0.6 vs. 1.2%).

## 4. Discussion

This investigation aimed to evaluate the challenges encountered and resources utilized by Austrian psychotherapists in the context of the ongoing pandemic. Furthermore, the study sought to discern the disparities in terms of challenges, resources, sociodemographic factors, and patterns of physical activity that typify psychotherapists exhibiting either good or poor well-being. To achieve this, responses to open-ended inquiries regarding perceived challenges and resources and a structured query on resource satisfaction were collected. The qualitative data obtained from open-ended questions were analyzed using conventional qualitative content analysis, subsequently categorizing the sample into two distinct groups, “Good WB” and “Poor WB,” based on their respective WHO-5 scores.

### 4.1. Comprehensive group burdens

Austrian psychotherapists reported facing numerous burdens, including mental health issues, a cumulation of global crises, COVID-19 restrictions, work-related strain, physical health concerns, dissatisfaction with societal development, financial worries, and uncertainty about the future.

“Mental health”-related suffering was the primary source of concern for psychotherapists during the COVID-19 pandemic. 71.7% of respondents reported grappling with negative emotions, exhaustion, overwhelming demands, and rumination. Among Austrian psychologists, even more individuals, namely 77.3% (24), acknowledged having experienced mental health issues. On the other hand, the general population mentioned mental health as the least prominent category (with less than 5% of the total sample) when inquired about sources of strain since the pandemic’s onset (19). Seemingly counterintuitive, the proportion of psychotherapists (16) exhibiting clinically significant mental health issues was notably lower than that of the general population. This finding suggests that psychotherapists displayed a heightened awareness of their mental well-being and considerable vigilance towards the negative repercussions of the pandemic and associated measures. This notion is corroborated by a prior study on the subjective perception of meaning among psychotherapists and patients in 2020, which revealed that physical and mental health was deemed more significant during the COVID-19 era than before (45). It can be assumed that this heightened awareness was the decisive factor activating various resources that contributed to the comparably positive mental health outcome of psychotherapists compared to the general population (16).

Interestingly, the second most crucial main category of burden reported by psychotherapists was the ongoing series of “global crises,” including the Russian aggression against Ukraine, the pandemic, and the climate crisis, as was endorsed by 37.4%. Within this category, the Ukraine war overshadows the pandemic due to our way of asking after the participants’ “primary current sources of burden.” At the time of our survey, the Austrian population was influenced by respective media reports. Notably, a smaller proportion of Austrian clinical psychologists, 26.7%, mentioned “global crises” as a burden, making it only their fourth most crucial category. Clinical psychologists’ second and third most essential burdens were related to more personal issues such as “work” and experienced “restrictions” due to COVID-19 measures. We attribute psychotherapists prioritizing global over personal concerns to two factors. On the one hand, the group of psychotherapists seems to enjoy a particular privilege over other health professions related to their work environment and social background, which is also reflected in the later discussed main category of “work” as a burden, which is endorsed by 6% more psychologists than psychotherapists and “work” a resource, which is endorsed by 10% more psychotherapists than clinical psychologists. Since psychotherapists mainly operate in private practice, part-time rather than full-time, we assume their work setting is characterized by relative autonomy and flexible time management. Research has demonstrated that, compared to salaried employees, self-employed individuals tend to exhibit a higher degree of satisfaction with their current occupations, particularly concerning the nature of their work (46). Moreover, psychotherapists are more likely than clinical psychologists to be socially selected because the entire training for becoming a psychotherapist, which includes hundreds of hours of training therapy, must be privately financed in Austria. Hence, the high training costs require a secure social background. Indeed, data from a cohort of 197 Austrian psychotherapy trainees revealed that most participants hailed from financially stable backgrounds and enjoyed satisfactory life circumstances (47). This combination of greater work autonomy and social selection might allow psychotherapists to focus more on global and abstract issues rather than personal problems.

The pandemic-induced “restrictions” constitute the third most frequently reported main category of burdens, embraced by 36.8% of the participating psychotherapists. Clinical psychologists scored similarly, with 33.1% (24). In contrast, this category was the most significant for the general population around the turn of 2020/2021 (19). This discrepancy likely results from the minimal containment measures in place during the survey of psychotherapists and clinical psychologists in spring 2022 and the fact that the question asking for burden refers to “current sources of burden,” that is, to the here and now in 2022, rendering a direct comparison with the general population study infeasible. At the time of the general population survey, a strict lockdown was enforced, causing responses to primarily focus on curfew measures and subsequent issues like reduced social contact, loneliness, and diminished cultural activities. Another aspect to consider is the impact of the lockdown on various economic sectors, and many people faced permanent or temporary leave or reduced working hours. Yet, the work of psychotherapists remained largely unaffected by stringent lockdown measures, with an even increased workload (9) compared to pre-pandemic data (48). While continuing one’s work during the pandemic could serve as a resource, the increased demand for mental healthcare services might also induce stress and exhaustion.

The present study supports this assumption, with 31.8% of psychotherapists acknowledging burdens related to “work.” A comparison of mental health professionals reveals that despite exhibiting similar concerns, psychotherapists are marginally less frequently impacted by work-related burdens than clinical psychologists, 37.8% of whom reported them (24). While not overstating this relatively minor discrepancy, this finding supports our argument that psychotherapists enjoy more adaptable working conditions. A striking 97.5% of psychotherapists operated in private practice, with only 2.5% working exclusively within institutional settings (16). Conversely, 74.4% of clinical psychologists also functioned in private practice, while 27.3% were employed in inpatient facilities (24). Since clinical psychologists work more frequently in institutional environments, they shoulder more administrative duties, coordinate more with other healthcare professionals, follow more strict and changing protocols, feel less valued and have less flexibility in managing their workload (35). The data further substantiate this notion, as more clinical psychologists than psychotherapists reported challenging working conditions (16.9 vs. 9.8%) and a difficult working atmosphere, including team conflicts (5.2 vs. 1.6%).

Other concerns psychotherapists raise include “physical health,” problems related to “family and friends,” “dissatisfaction with societal development,” “financial concerns,” and concerns about an “uncertain future.” Considering the current high inflation, the mere 9.2% of psychotherapists expressing financial concerns confirms that this group predominantly comprises individuals with satisfactory financial life situations. The protective role of economic security on mental health is reinforced by multivariable analyses performed on a representative sample of the Austrian general population surveyed in April 2022. These analyses demonstrated that, among various sociodemographic factors, household income had the strongest association with mental health (49).

Concluding the analysis on self-reported burdens among psychotherapists, it is worth mentioning that a slightly higher proportion of psychotherapists, namely 17.5%, experienced no or only minor adverse impacts compared to psychologists at 12.8%, suggesting a slightly lesser impact of the pandemic’s ramifications on psychotherapists. This difference, although it should not be overstated, again points to slightly greater resilience of psychotherapists due to the already mentioned factors, such as high work autonomy, satisfaction typical for self-employed individuals (46) and a secure social background (47). The selective nature of the admission process for psychotherapists’ specialized training could also be a further contributing factor. Roughly 25% of applicants for becoming a psychotherapist discontinue their training following the first phase (step one) and fail to proceed to the specialized training segment (step two). The program mandates significant introspection and training therapy to guarantee the persistence of candidates demonstrating a high degree of reflective competence. Research has shown that enhanced reflective capacity is linked to deliberate efforts to cultivate it, a secure environment, the support of peers, and allocated time for reflection (50).

### 4.2. Comprehensive group resources

Regarding stress-coping resources, psychotherapists were observed to primarily employ active coping strategies, such as seeking “social contacts,” practicing “mindfulness “, partaking in “recreational activities,” finding joy in “working” and engaging in “inner processes” such as cultivating a positive attitude. These strategies are correlated with reduced psychological distress (26–29) and stress symptoms (51) among mental health professionals. A discernible positive impact may explain why over a third of the respondents expressed being “very satisfied,” and nearly half conveyed at least “satisfied” with the coping strategies they employed to navigate challenges.

Remarkably, the most robust set of resources mentioned is highly similar between psychotherapists, clinical psychologists (24) and the general population (19). In all three cohorts, “social contacts” as a resource achieved the highest overall score among all resources cited. Prior research substantiates the role of social bonds in alleviating mental health symptoms during the pandemic (52–54). In a review encompassing 31 studies on the coping behaviors of healthcare workers, Labrague et al. (55) identified support from and communication with family, friends, and colleagues as a primary coping mechanism for addressing the adverse ramifications of the COVID-19 pandemic.

The second most prominent concern for psychotherapists revolved around “mindfulness.” The category encompasses practices seamlessly interwoven into daily life, such as “slowing down,” focusing,” and “self-care,” in addition to “mental techniques and exercises” like yoga, breathwork meditation, and gratitude. Like clinical psychologists (24), psychotherapists were particularly forthcoming in enumerating various mindfulness approaches, perhaps attributable to their professional expertise. Previous research underscores the potential of mindfulness practices in bolstering resilience and fortifying one’s capacity to navigate adversity during crises (56–58).

“Recreational activities” emerged as the third most frequently cited category by psychotherapists but the second among clinical psychologists and the Austrian general population surveyed during the winter of 2020/2021. This category encompassed activities such as immersing oneself in nature, engaging in sports, and discovering new or pursuing hobbies. Physical activity was identified as a resource by 23.2% of psychotherapists and 22% of clinical psychologists (24), in contrast to a mere 11% of the general population (19). The significance of physical activity for mental health has been emphasized in numerous prior studies (59, 60). Moreover, a study conducted on a representative sample of the Austrian general population in April 2022 revealed heightened odds of experiencing depressive symptoms, anxiety, insomnia, stress, alcohol abuse, and eating disorders among physically inactive individuals compared to their active counterparts (61).

“Work” as a resource also surfaced for 38.4% of the psychotherapists, compared to nearly a third of the clinical psychologists surveyed (24), starkly contrasting to the mere 5% of the general population in winter 2020/2021 (19). Multiple factors account for the prevalence of professional references among psychotherapists. Prior research indicates that assisting others can foster one’s ability to cope with crises (62, 63). As such, helping patients navigate the pandemic might have equipped psychotherapists with a valuable resource for managing their own well-being. Also, the pandemic-induced shifts in their work environment—such as enhanced flexibility due to remote work and even virtual patient care—may have played a role. In fact, 19.1% of psychotherapists identified digital home-based work as a favorable outcome of the pandemic. Additionally, the widespread mental health strain heightened the perceived importance of mental healthcare services among policymakers, the media, and society at large. Consequently, mental health professionals might have experienced a surge in job-related meaning, a known safeguard against occupational stress and related mental health disorders (62, 63). The fact that more psychotherapists than clinical psychologists endorsed “work” as a resource might have to do with those mentioned more liberal and satisfactory working conditions of psychotherapists.

Over a third of psychotherapists, just like clinical psychologists, identified “internal processes,” such as “a positive attitude “, “resilience,” and “self-reflection” as vital resources. A study on the Austrian general population during the first COVID-19 lockdown substantiates the protective function of a positive mindset (28), revealing its association with reduced stress, depression, anxiety, and sleeplessness. Psychotherapists’ affirmative outlooks are further evidenced by the scant 12.1% of participants who found no positive elements related to the pandemic. Waters et al. proposed a dynamic interplay between positive emotions and psychological distress, asserting that such emotions mitigate mental health risks, preserve mental well-being, and facilitate the transformation of crises into opportunities for novel insights or tactics (64). Our study’s written reports demonstrate that, akin to respondents in Yang et al.’s interviews (65), Austrian psychotherapists, like clinical psychologists, employed positive coping strategies such as refocusing and reappraisal.

### 4.3. Group characteristics “Good WB” group vs. “Poor WB”

Notable differences were observed in the sample when split into a Good Well-Being (“Good WB”) and Poor Well-Being (“Poor WB”) group, partitioned based on levels of psychological well-being (44).

#### 4.3.1. Sociodemographic factors

The “Poor WB” group consisted of more females. It was younger, less experienced, with fewer psychotherapists in the age groups 60–69 and 70+ and more in the age groups 41–49 and 50–59. The “Good WB” group” on the other hand, reported participating more frequently (4+ days/week) in physical activity compared to the “Poor WB” group (0–2 days/week). This disparity is especially pronounced when considering the proportion of psychotherapists who abstain from physical activity entirely: a mere 5.6% from the “Good WB” group, as opposed to a significant 14.6% within the “Poor WB” group. An overrepresentation of women, younger individuals, and physically inactive persons within a cohort exhibiting poor well-being aligns with prior findings from the general population, suggesting that the pandemic has particularly affected these demographics (2, 49). In our sample, the higher burden on women between 40 and 60 is likely related to their increased care workload during the pandemic (66, 67). Given the legal stipulation in Austria that requires psychotherapists to be over 24 to commence the second phase of their training (68), it is likely that many psychotherapists who participated in this survey were already middle-aged as they found themselves navigating the delicate balance between childcare obligations, a demanding workload, and other pandemic-related stressors. In line with our findings regarding age and work experience, a systematic review also found that an increase in age and work experience is related to decreased reported stress among mental health professionals (69).

As we anticipated, the disparity between the groups also appears to stem from differing work settings, namely the private practice vs. the institutionalized setting. A higher proportion of the “Good WB” group (80.4%) worked exclusively in private practice compared to the “Poor WB” group (71.9%). Conversely, a greater percentage of psychotherapists in the “Poor WB” group (28.1%) were employed by institutions, in contrast to the “Good WB” group (19.6%). This observation seems to corroborate our prior assumptions regarding what makes “work” a frequently cited resource and a less frequently cited burden for psychotherapists (compared to clinical psychologists) and what gives them the freedom to engage in global problems (such as the climate crisis or the Russian war against Ukraine) rather than more personal problems. The enhanced flexibility of working conditions for self-employed professionals, characterized by a higher degree of autonomy and adaptable time management, contrasts with the more restrictive conditions and high workloads of institutional environments during a pandemic (35, 36), thereby contributing to improved well-being. A larger number of weekly patients (19.13, SD = 9.27 vs. 16.98, SD = 8.88, *p* = 0.013) also seems to have bolstered – or was an expression of – better well-being of psychotherapists. One potential explanation could be that the “Good WB” group, perhaps due to fewer care responsibilities and a more liberal work setting with reduced administrative tasks, had more time to treat patients, which might have felt gratifying (62, 63).

#### 4.3.2. Burdens

Regarding the burdens faced, psychotherapists belonging to the “Poor WB” group encountered heightened “interpersonal difficulties” and a more pronounced strain across various mental health realms, such as “excessive demand,” “negative emotions,” “sleep disturbances,” “fatigue,” “physical health,” and “somatic complaints.” Notably, 40.4% of participants within the “Poor WB” group reported somatic complaints, which doubles the frequency observed in the “Good WB” group. The reported somatic complaints encompass not only those typically categorized as psychosomatic such as tense muscles, back pain, tinnitus or headaches but also chronic physical ailments, age-related issues, and more. Unsurprisingly, the “Poor WB” group also declared significantly less often to have experienced “no or little adverse impacts” of the pandemic (2.9 vs. 26.6%).

We think that this pattern points not only to more individuals with poorly integrated personalities in the “Poor WB” group but also to more individuals suffering from chronic illness, age-related ailments and other factors related to the demographics specific to this group, such as the burden of care typical for the age group between 40 and 60 or a more detrimental work environment as pointed out above in the demographics section.

#### 4.3.3. Resources

In examining the resources utilized, the “Good WB” group intriguingly reported a less frequent reliance on mindfulness, potentially due to a diminished need for coping. This notion is reinforced by their significantly higher “positive attitude/optimism” and “increased importance of health,” suggesting that they were able to perceive constructive aspects in the containment measures rather than solely regarding them as burdensome.

The “Good WB” group also reported participating in recreational activities less frequently, especially “physical activity.” This observation contradicts the previously mentioned finding that the “Good WB” group engaged in “physical activity” considerably more often (4+ days/week) compared to the “Poor WB” group (0–2 days/week). It is plausible that they reported “physical activity” more frequently due to discovering sports as a resource only during the pandemic, while the “Good WB” group, having consistently engaged in physical activity prior to the pandemic, may have deemed physical activity less noteworthy to report. The reduced propensity for *de facto* physical involvement among the “Poor WB” group could be ascribed to their struggles in various mental and physical health domains. Possibly due to a combination of overall better well-being and greater ease in accessing helpful resources, particularly physical activity, the “Good WB” group reported higher satisfaction with their coping strategies, with nearly half being “very satisfied” compared to only one-fifth in the “Poor WB” group.

### 4.4. Limitations

This study presents several limitations. Firstly, the written format of the study constrains the potential to glean more contextually rich and coherent information, as would be feasible through personal interviews. Secondly, all questions were posed during a period of fewer pandemic-related restrictions, potentially leading to recall bias when inquiring about the challenges and resources experienced throughout the pandemic. Additionally, we did not distinguish timeframes in the burdens category, even though questions one and two pertain to the present, and question three relates to the entire pandemic period. Thirdly, other crises, such as the war in Europe and corresponding high inflation rates, likely influenced the reported burdens and resources. Fourth, physical activity was not assessed objectively but rather by one self-report question. Fifth, the WHO-5 scale is a global measure that does not capture nuanced experiences of stress or specific psychiatric constructs such as anxiety or depression. With regard to our comparison of psychotherapists with clinical psychologists (24), we would like to bring to attention that psychotherapists are older (53 vs. 45 years) and have a lower proportion of women (81 vs. 92%) compared to clinical psychologists, which reduces comparability. Comparability is also reduced by the fact that 139 of 513 psychotherapists also possessed clinical psychology training, though only 74 were actively working in the field of clinical psychology.

## 5. Conclusion

Psychotherapists identified mental health-related phenomena as their primary source of burden, suggesting heightened awareness of their own declining psychological well-being. Global crises, notably influenced by the war in Ukraine, represented the second most significant category of burden, overshadowing complications brought about by the pandemic. However, the pandemic’s repercussions, particularly discomfort from containment measures, emerged as the third principal stressor. The key resources for managing these challenges were social connections, mindfulness, work fulfilment, and internal processes. Notably, individuals with better well-being were characterized by increased physical activity, older age, more years of professional experience, a lower ratio of females, being self-employed in private practice rather than employed in institutional settings, and handling a higher patient caseload compared to the group with poor well-being. This “better well-being” group also tended to exhibit a more optimistic outlook, a greater focus on maintaining good health, and higher satisfaction with their own coping methods. Our findings underscore the potential for shaping more effective support systems, policies, and educational programs that bolster the resilience of mental health professionals amidst global crises. Moreover, they highlight strategies that individual practitioners can adopt to preserve their own well-being during challenging times.

## Data availability statement

The raw data supporting the conclusions of this article will be made available by the authors, without undue reservation.

## Ethics statement

The studies involving humans were approved by Ethical number: E.K. G.Z. 11/2021–2024. The studies were conducted in accordance with the local legislation and institutional requirements. The participants provided their written informed consent to participate in this study.

## Author contributions

YS, AJ, EH, and TP: conceptualization. YS: methodology, validation, and writing—original draft preparation. MB, BS, YS, and EH: formal analysis. EH: investigation, data curation, and project administration. YS, EH, AJ, TP, and CP: writing—review and editing. YS and EH: visualization and supervision. All authors contributed to the article and approved the submitted version.

## Conflict of interest

The authors declare that the research was conducted in the absence of any commercial or financial relationships that could be construed as a potential conflict of interest.

## Publisher’s note

All claims expressed in this article are solely those of the authors and do not necessarily represent those of their affiliated organizations, or those of the publisher, the editors and the reviewers. Any product that may be evaluated in this article, or claim that may be made by its manufacturer, is not guaranteed or endorsed by the publisher.
