# Supplementary material for: Understanding pandemic resilience: a mixed-methods exploration of burdens, resources, and determinants of good or poor well-being among Austrian psychotherapists
Source: Front Public Health. 2023 Aug 24;11:1216833. doi: 10.3389/fpubh.2023.1216833 (PMC10483144; doi:10.3389/fpubh.2023.1216833)
Supplement: Supplementary file 1 [file Table_1.pdf]

Supplementary Material

Understanding Pandemic Resilience: A Mixed-Methods Exploration of Burdens, Resources, and Determinants of Good or Poor Well-being Among Austrian Psychotherapists

1. Supplementary Tables

Suppl. Table 1 Proportion of codings[1] to describe one or more burdens in each of the main and subcategories by group (n = 513)

Category		Group: Good Wellbeing (n = 342)	Group: Poor Wellbeing (n = 171)	Statistics
Catastrophes	%	54.4%	50.3%	χ^2 (1) = 0.767;
	n	186	86	*p* = 0.381
climate crisis	%	8.5%	7.0%	χ^2 (1) = 0.331;
	n	29	12	*p* = 0.565
pandemic	%	9.6%	13.5%	χ^2 (1) = 1.694;
	n	33	23	*p* = 0.193
refugee crisis	%	1.2%	0.6%	χ^2 (1) = 0.404;
	n	4	1	*p* = 0.525
war	%	35.4%	29.2%	χ^2 (1) = 1.934;
	n	121	50	*p* = 0.164
Finances	%	9.4%	11.7%	χ^2 (1) = 0.685;
	n	32	20	*p* = 0.408
being underpaid	%	1.2%	2.9%	χ^2 (1) = 2.036;
	n	4	5	*p* = 0.154
general worries	%	4.7%	4.1%	χ^2 (1) = 0.091;
	n	16	7	*p* = 0.763
inflation	%	3.5%	4.7%	χ^2 (1) = 0.416;
	n	12	8	*p* = 0.519
Friends and Family	%	20.2%	26.3%	χ^2 (1) = 2.487;
	n	69	45	*p* = 0.115
**interpersonal problems**	**%**	**15.2%**	**24.6%**	**χ^2 (1) = 6.669;**
	**n**	**52**	**42**	***p* = 0.010**
problems with children	%	5.0%	1.8%	χ^2 (1) = 3.148;
	n	17	3	*p* = 0.076
**Mental health**	**%**			**χ^2 (1) =;**
	**n**			***p* =**
depressive mood	%	2.9%	6.4%	χ^2 (1) = 3.575;
	n	10	11	*p* = 0.059
**excessive demand**	**%**	**15.5%**	**24.6%**	**χ^2 (1) = 6.207;**
	**n**	**53**	**42**	***p* = 0.013**
mental health of others	%	2.0%	1.80%	χ^2 (1) = 0.051;
	n	7	3	*p* = 0.821
**negative feelings**	**%**	**28.7%**	**50.9%**	**χ^2 (1) = 24.416;**

[1] While a subcategory was assigned to a study participant at most once, and thus the number of subcategories corresponds to the number of study participants who reported them, this is not necessarily the case for the main categories. A participant who reported several subcategories (e.g. sleep and negative feelings) was counted multiple times in a main category (e.g. mental health).

	**n**	**98**	**87**	**$p < 0.001$**
**problems with sleep**	**%**	**9.6%**	**18.7%**	**χ^2 (1) = 8.465;**
	**n**	**33**	**32**	**$p = 0.004$**
rumination	%	16.7%	15.8%	χ^2 (1) = 0.064;
	n	57	27	$p = 0.800$
**symptoms of exhaustion**	**%**	**11.7%**	**33.3%**	**χ^2 (1) = 43.809;**
	**n**	**40**	**57**	**$p < 0.001$**
**Physical health**	**%**	**28.7%**	**46.8%**	**χ^2 (1) = 16.535;**
	**n**	**98**	**80**	**$p < 0.001$**
physical health of others	%	9.6%	7.0%	χ^2 (1) = 0.987;
	n	33	12	$p = 0.321$
**somatic complaints**	**%**	**19.0%**	**40.4%**	**χ^2 (1) = 26.915;**
	**n**	**65**	**69**	**$p < 0.001$**
Work	%	34.8%	43.3%	χ^2 (1) = 3.493;
	n	119	74	$p = 0.062$
working atmosphere	%	1.2%	2.9%	χ^2 (1) = 2.036;
	n	4	5	$p = 0.154$
fewer clients	%	1.5%	0.6%	χ^2 (1) =0.759;
	n	5	1	$p = 0.384$
high workload	%	24.6%	30.4%	χ^2 (1) = 2.001;
	n	84	52	$p = 0.157$
organisational framework	%	9.6%	10.5%	χ^2 (1) = 0.098;
	n	33	18	$p = 0.754$
Dissatisfaction with societal development	%	17.0%	17.5%	χ^2 (1) = 0.027;
	n	58	30	$p = 0.868$
general societal development	%	8.8%	8.2%	χ^2 (1) = 0.050;
	n	30	14	$p = 0.824$
politics and media reporting	%	9.1%	9.9%	χ^2 (1) = 0.103;
	n	31	17	$p = 0.748$
**No answer**	**%**	**37.1%**	**23.4%**	**χ^2 (1) = 9.806;**
	**n**	**127**	**40**	**$p = 0.002$**
**No or little negative effects**	**%**	**26.6%**	**2.9**	**χ^2 (1) = 42.039;**
	**n**	**91**	**5**	**$p < 0.001$**
Not well classifiable	%	7.3%	8.2%	χ^2 (1) = 0.125;
	n	25	14	$p = 0.724$
Restrictions	%	43.3%	49.7%	χ^2 (1) = 1.903;
	n	148	85	$p = 0.168$
Unknown future	%	13.7%	12.3%	χ^2 (1) = 0.212;
	n	47	21	$p = 0.645$

p: p -values (2-tailed); χ^2: Chi-squared-test.

Suppl. Table 2 Proportion of codings[2] to describe one or more resources in each of the main and subcategories by group (n = 513)

		Group		
Category		Good Wellbeing (n = 342)	Poor Wellbeing (n = 171)	Statistics
Health	%	7.3%	5.8%	χ^2 (1) = 0.383;
	n	25	10	$p = 0.536$

[2] While a subcategory was assigned to a study participant at most once, and thus the number of subcategories corresponds to the number of study participants who reported them, this is not necessarily the case for the main categories. A participant who reported several subcategories (e.g. hobbies and physical activity) was counted multiple times in a main category (e.g. recreational activity).

having sought professional help related to health	%	3.2%	5.8%	χ^2 (1) = 2.011;
	n	11	10	*p* = 0.156
**increased importance of health**	**%**	**4.1%**	**0%**	**χ^2 (1) = 7.196;**
	**n**	**14**	**0**	***p* = 0.007**
**Inner Processes**	**%**	**43.6%**	**34.5%**	**χ^2 (1) = 3.886;**
	**n**	**149**	**59**	***p* = 0.049**
**positive attitude/optimism**	**%**	**19.6%**	**10.5%**	**χ^2 (1) = 6.776;**
	**n**	**67**	**18**	***p* = 0.009**
resilience	%	14.3%	10.5%	χ^2 (1) = 1.451;
	n	49	18	p = 0.228
resistance	%	1.2%	4.1%	χ^2 (1) = 1.451;
	n	4	7	*p* = 0.228
self-reflection	%	8.8%	9.4%	χ^2 (1) = 0.048;
	n	30	16	*p* = 0.827
**Mindfulness**	**%**	**78.7%**	**87.1%**	**χ^2 (1) = 5.432;**
	**n**	**269**	**149**	***p* = 0.020**
prioritizing/rethinking of values	%	17.3%	21.1%	χ^2 (1) = 1.092;
	n	59	36	*p* = 0.296
acceptance	%	1.8%	1.8%	χ^2 (1) = 0.000;
	n	6	3	*p* = 1.000
gratitude	%	2.3%	4.7%	χ^2 (1) = 2.064;
	n	8	8	*p* = 0.151
mental techniques and exercises	%	11.4%	14.6%	χ^2 (1) = 1.080;
	n	39	25	*p* = 0.299
reduction of media consumption	%	3.8%	3.5%	χ^2 (1) = 0.027;
	n	13	6	*p* = 0.869
religion/spirituality	%	2.3%	2.9%	χ^2 (1) = 0.158;
	n	8	5	*p* = 0.691
self-care	%	14.0%	17.0%	χ^2 (1) = 0.764;
	n	48	29	*p* = 0.382
slowing down	%	26.0%	21.6%	χ^2 (1) = 1.184;
	n	89	37	*p* = 0.277
Other resources	%	10.2%	12.9%	χ^2 (1) = 0.799;
	n	35	22	*p* = 0.371
increase in financial resources	%	2.0%	0.6%	χ^2 (1) = 1.587;
	n	7	1	*p* = 0.208
resources from vacations	%	3.2%	7.0%	χ^2 (1) = 3.846;
	n	11	12	*p* = 0.050
structure, routine, self-organization in private and professional life	%	5%	5.3%	χ^2 (1) = 0.020;
	n	17	9	*p* = 0.887
**Recreational activities**	**%**	**53.8%**	**73.1%**	**χ^2 (1) = 17.725;**
	**n**	**184**	**125**	***p* < 0.001**
being outdoors	%	20.8%	25.1%	χ^2 (1) = 1.269;
	n	71	43	*p* = 0.260
hobbies	%	12.9%	17.0%	χ^2 (1) = 1.565;
	n	44	29	*p* = 0.211
**Physical activity**	**%**	**20.5%**	**31.0%**	**χ^2 (1) = 6.930;**
	**n**	**70**	**53**	***p* = 0.008**
**Satisfaction with coping**				**χ^2 (4) = 55.443;**
**very satisfied**	**%**	**43.4%**	**19.6%**	***p* < 0.001**
	**n**	**145**	**33**	
**satisfied**	**%**	**48.2%**	**48.8%**	
	**n**	**161**	**82**	
**neutral**	**%**	**6.6%**	**25.6%**	
	**n**	**22**	**43**	
**dissatisfied**	**%**	**1.2%**	**4.8%**	
	**n**	**4**	**8**	
**very dissatisfied**	**%**	**0.6%**	**1.2%**	

	**n**	**2**	**2**	
Social contacts	%	77.8%	78.9%	χ^2 (1) = 0.091;
	n	266	135	p = 0.762
colleagues	%	4.7%	5.3%	χ^2 (1) = 0.084;
	n	16	9	p = 0.772
fewer social contacts and	%	7.6%	7.6%	χ^2 (1) = 0.000;
obligations	n	26	13	p = 1.000
other social contact	%	16.4%	15.2%	χ^2 (1) = 0.116;
	n	56	26	p = 0.733
partners, family, and friends	%	44.2%	49.1%	χ^2 (1) = 1.135;
	n	151	84	p = 0.287
pets	%	5.3%	2.9%	χ^2 (1) = 1.457;
	n	18	5	p = 0.227
Work	%	45.3%	45.6%	χ^2 (1) = 0.004;
	n	155	78	p = 0.950
flexible working conditions	%	20.2%	18.7%	χ^2 (1) = 0.154;
	n	69	32	p = 0.695
less work	%	4.7%	4.1%	χ^2 (1) = 0.091;
	n	16	7	p = 0.763
recognition for psychosocial	%	2.6%	1.2%	χ^2 (1) = 1.161;
services	n	9	2	p = 0.281
supervision/intervision	%	6.1%	4.7%	χ^2 (1) = 0.457;
	n	21	8	p = 0.499
working (in itself)	%	12.0%	17.0%	χ^2 (1) = 2.390;
	n	41	29	p = 0.122
no answer	%	25.1%	22.8%	χ^2 (1) = 0.338;
	n	86	39	p = 0.561
no positive effects	%	10.8%	16.4%	χ^2 (1) = 3.180;
	n	37	28	p = 0.075
not well classifiable	%	5.8%	3.5%	χ^2 (1) = 1.296;
	n	20	6	p = 0.255

p: p -values (2-tailed); χ^2: Chi-squared-test.
